# Discrimination of Inner Injury of Korla Fragrant Pear Based on Multi-Electrical Parameters

**DOI:** 10.3390/foods12091805

**Published:** 2023-04-26

**Authors:** Jing An, Xiuzhi Luo, Lijian Xiong, Xiuying Tang, Haipeng Lan

**Affiliations:** 1 College of Engineering, China Agricultural University, Beijing 100083, China; 2 College of Mechanical and Electrical Engineering, Tarim University, Alar 843300, China

**Keywords:** Korla fragrant pear, inner injury, electrical characteristic parameters, support vector machine, correlation analysis, principal component analysis, correlation analysis

## Abstract

Inner-injury fragrant pears are easily prone to rot during storage. Discriminating inner injury in the Korla fragrant pear from the normal pear is difficult as the flesh may be injured while the peel of the fruit remains intact. This study demonstrated the recognition of inner-injury pears based on their electric characteristics to pick out the inner-injury pears before storage. The electrical parameters parallel equivalent capacitance, quality factor, parallel equivalent inductance, parallel equivalent resistance, complex impedance, and phase angle were measured using the fruit electrical characteristic detection instrument. Principal component analysis and correlation analysis were used to determine the characteristic parameters, connected with the qualitative value of the fragrant pear to establish three discrimination models. When the measurement frequency was 100 kHz, compared with the Naïve Bayes and K-nearest neighbor models, the Support Vector Machine model with the characteristic parameters of quality factor, parallel equivalent resistance, and phase angle performed best. The recognition accuracy of the test set was 92.00%, the precision was 92.41%, the recall was 97.33%, and the F_1_ score was 0.95. Therefore, the electrical characteristic technique effectively detected the inner injury of fragrant pears and provided a new way to distinguish the inner injury of fruits.

## 1. Introduction

Korla fragrant pears are hand-harvested, graded, and packaged. Following harvesting, 85% of the pears are stored and sold at the appropriate time [1]. Fragrant pear has a thin pericarp and crispy sarcocarp, and improper handling would cause irreversible injury to the pear. The appearance of inner-injury fragrant pears is identical to that of normal fragrant pears, and they can be stored in the same manner. This part of fragrant pears is perishable during the storage period [2]. If not removed promptly after decay, it will infect other fragrant pears, resulting in greater losses. To reduce the economic losses of storage enterprises, inner-injury pears should be sorted out before storage and sold in grades or used as raw materials for pear processing products. Therefore, it is of great significance to use a non-destructive and accurate method to detect inner-injury fragrant pears for storage enterprises.

In recent years, researchers have mainly used hyperspectral imaging [3,4,5,6,7,8,9], machine vision [10,11], thermal imaging [12], nuclear magnetic resonance [13], electronic nose [14], multispectral reflection, and fluorescence imaging [9] to detect mechanical damage, bruise, and the internal quality of fruit. However, these technologies are limited in practical application due to the high equipment costs, long data acquisition and processing time, and possible health and safety issues [15]. Therefore, a fast and reliable method for detecting fruit injury is required.

The electrical properties’ detection technology has the characteristics of being fast, simple, and non-destructive, and is increasingly used in the detection of agricultural products [16,17]. Fruit is a weakly conductive medium, with a large number of charged particles inside. The amount and spatial distribution of electric charge in fruit change correspondingly with the metabolic activities of tissues and cells [18]; the change in the internal organizational structure of fruits would cause a change in physiological state, which would be reflected in the change in dielectric properties [19]. Lan et al. combined electrical parameters with the internal quality of fruit at different measurement frequencies to determine the soluble solid content of fruit [20,21,22,23]; Guo et al. judged the maturity of apples by using dielectric spectroscopy [24,25]. It was feasible to use electrical characteristics to detect fruit damage. Tu et al. predicted the volume of apple damage based on dielectric properties at a measurement frequency of 1 MHz [26]. zhang et al. achieved good results by detecting the quality of damaged Korla fragrant pears using electrical characteristic parameters at 1 kHz [27]. Currently, electrical characterization techniques have been used to detect internal components and dominant damage in fruits. However, it is not known whether the electrical parameters can be used to detect the inner injury of Korla fragrant pear, and which measurement frequency is more suitable to identify the inner injury. This paper discussed the method of detecting the inner injury of Korla fragrant pear based on the bioelectrical characteristics.

Taking all of these factors into consideration, the specific research objectives of this study included the following aspects: (1) to obtain the electrical parameters of fragrant pear using electrical parameter acquisition equipment at three different measurement frequencies; (2) to obtain the characteristic electrical parameters and the optimal measurement frequency for identifying the inner injury; (3) to establish classification models to distinguish the inner injury and normal fragrant pear.

## 2. Materials and Methods

### 2.1. Korla Fragrant Pear

The Korla fragrant pears used in this study were collected from the Jiutuan Sanlian conventional management pear orchard (40°34′32″ N, 81°13′11″ E) in Alar City, a high-quality fragrant pear production area in southern Xinjiang, wherew the trees’ age is 12 years. Fragrant pears with regular shapes, uniform fruit sizes, similar colors, and no defects were randomly picked from the pear orchard and transported to the laboratory on the same day. The selected fragrant pears with a weight of 120 g ± 10 g, diameter range of 58 ± 4 mm at the equator, normal color, and regular appearance were selected as test samples. The fragrant pears were gently wiped clean and placed for 12 h in the laboratory to stabilize the fragrant pear physiological changes.

### 2.2. Inner-Injury Samples

The pre-experiment found that the visible injury occurred when the static pressure deformation was 8 mm, and the threshold of inner-injury deformation was set to 7 mm. The very small inner injury had little effect on the quality of the fragrant pear [2]. Considering the different degrees of inner injury in actual production, the static pressure deformation was set as 3 mm, 5 mm, and 7 mm, representing the whole range of inner injury. Every deformation group had 100 samples. The other 100 samples were set as the control group without static pressure, namely, the static pressure deformation was 0 mm.

The inner-injury samples were made on the universal testing machine (WD-D3-7, HST, Jinan, China), and the loading speed was set to 20 mm/min. The fragrant pears were placed horizontally on the lower circular indenter of the experimental platform. When the universal testing machine was started up, the upper circular indenter slowed down until it contacted the equatorial position of the fragrant pear, slowly pressurized. Participants in the experiment were equipped with cotton gloves from picking pears to the end of the experiment to prevent the pears from being damaged.

### 2.3. Experimental Equipment and Data Acquisition

**Figure 1:**
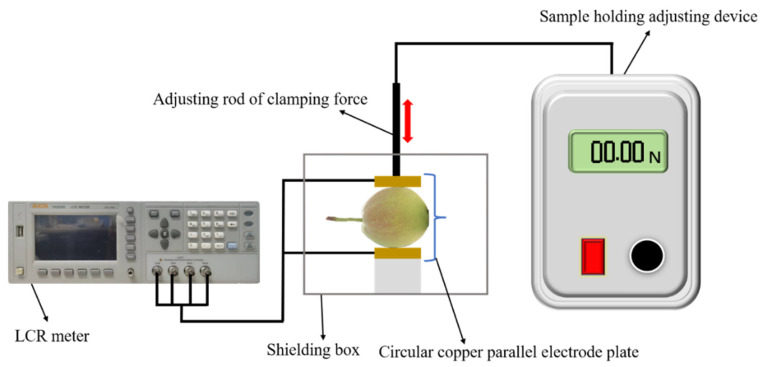
Electrical parameter measurement system for Korla fragrant pears.

Electrical parameter acquisition equipment mainly included an LCR digital bridge, parallel plate electrode, shielding box, and sample holding adjustment device, as shown in Figure 1. The LCR digital bridge model was TH2828s (Tonghui Electronics Co., Ltd., Changzhou, China), with a measurement frequency range of 20 Hz~1 MHz. The electrode was a circular copper parallel plate electrode with a diameter of 30 mm.

The air between the polar plates and the surface of Korla fragrant pear may affect the measurement results. A pair of conductive sponges were placed on the surface of the upper and lower polar plates, and Korla pear was sandwiched between the conductive sponges of the two plates. When the preload holding force is greater than 1.5 N, it has a great influence on electrical parameters. In this study, a preload of 1.0 N was used [28]. The electrodes were connected to the digital bridge via a coaxial cable through a four-terminal pair configuration, and the measurement results were displayed on the bridge screen and then recorded.

### 2.4. Circuit Model

Fruit cells had electrical properties similar to resistance and capacitance. When the direct current was applied to it, all the current passed through the resistance due to the infinite capacitive reactance of the capacitor. However, if the alternating current was applied, most of the current that passed through the capacitor and flowed through varied with the frequency. Agricultural materials might be viewed as an equivalent circuit made of capacitance and resistance in series and parallel based on the properties of capacitance and resistance [29].

Series and parallel equivalent circuit configurations are available for the LCR’s electrical parameter measurement circuit. For the most part, a series equivalent circuit was employed to test low-impedance elements with a value lower than 100 Ω, while a parallel equivalent circuit was employed to test high-impedance components [30]. The impedance values measured in this experiment were more than 100 Ω, and the parallel equivalent circuit was used to measure the electrical parameters.

The frequencies were set to 10 kHz, 100 kHz, and 1 MHz, while the measuring voltage was set to 1 V. As the internal medium of the capacitor, a fragrant pear sample was positioned between the electrodes. The driving current output by the sine wave generator flows through a circuit formed of fruit as the capacitor medium. The electrical parameters equivalent capacitance (Cp), quality factor (Q), equivalent resistance (Rp), complex impedance (Z), parallel equivalent inductance (Lp), and phase angle (θ) of fragrant pear were measured.

### 2.5. Data Processing and Analysis

The electrical parameters were subjected to principal component analysis and correlation analysis to determine the best modeling parameters. Origin 2019b (OriginLab Corporation, Northampton, MA, USA) was used to analyze the data and depict the analysis results.

A correlation between variables can be understood as a particular amount of information overlap between variables. Principal Component Analysis (PCA) is the process of replacing duplicate variables (related variables) with extracted features in all variables, creating as few new variables as possible, making these new variables irrelevant, and retaining as much original information as possible when reflecting target information [31]. By modeling utilizing features taken from main components as input parameters, noise and interference from redundant information may decrease, and the model’s ability to predict outcomes might be improved [32].

The correlation analysis method was utilized to examine the relationship between electrical parameters as well as the relationship between electrical parameters and category labels. To describe the degree of correlation between electrical measurements and category labels, the correlation coefficient r was used. A negative correlation was denoted by a negative value, whereas a positive correlation was indicated by a positive number. The stronger the correlation, the larger the absolute value of the correlation coefficient. |r| ≤ 0.1 indicated no correlation, 0.1 < |r| < 0.4 indicated a weak linear correlation, 0.4 < |r| < 0.7 indicated a medium linear correlation, 0.7 < |r| < 0.9 indicated a strong correlation, and |r| > 0.9 indicated a highly correlated relationship [33].

### 2.6. Classification Modeling Method

It is a binary classification issue to distinguish inner-injury pear from normal pear. Naïve Bayes (NB), K-nearest neighbor (KNN), and Support Vector Machine (SVM) were all popular classification approaches. Cover and Hart first proposed the K-nearest neighbor approach in 1968 [34]. They believed that proximity samples had similar categories, and their classification was based on the distance between the closest k samples in the feature space. Naïve Bayes was an algorithm that used the Bayesian theorem to classify small- or medium-sized data [35]. SVM was a learning classification method proposed by Cortes and Vapnik based on statistical theory [36]. It was a binary classification model that constructed the optimal hyperplane with the maximum classification interval to solve classification problems, with the characteristics of fewer redundant samples and good robustness.

Depending on whether or not the samples were subjected to pressure, they were labeled 0 or 1. After static pressure treatment, 0 represented normal pear and 1 represented inner-injury pear. The non-destructive pear and the inner-injury pear were randomly divided into the training and the test sets according to the ratio of 3:1. A training set of 300 samples was used for modeling, and a set of 100 samples was used to test the models’ discrimination ability. The modeling and model validation processes were completed with the software Matlab2019a (Mathworks Inc., Natick, MA, USA).

### 2.7. Model Evaluation

The performance of the NB, SVM, and KNN classification models for recognition was assessed using accuracy, precision, recall, and F_1_ score [37]. For the binary classification problem, samples can be divided into true positive (TP), false positive (FP), true negative (TN), and false negative (FN) according to the combination of the true and predicted categories. If *TP*, *FP*, *TN*, and *FN* represent the corresponding number of samples, then TP+FP+TN+FN= total number of samples. Accuracy is the ratio of correct classification, defined as:

(1) $$\mathrm{Accuracy}=\frac{TP+TN}{TP+TN+FP+FN}$$

Precision is the ratio of actual positives to the total predicted positives, defined as:

(2) $$\mathrm{Precision}=\frac{TP}{TP+FP}$$

Recall is the ratio of true positives to total actual positives in the data, defined as:

(3) $$\mathrm{Recall}=\frac{TP}{TP+FN}$$

F_1_ score is defined as the harmonic mean of precision and recall is a single summary measure of a classifier’s performance.

(4) $$F_{1}=\frac{2*\mathrm{Precision}*\mathrm{Recall}}{\mathrm{Precision}+\mathrm{Recall}}$$

In this study, the samples of inner-injury fragrant pears were designated as the positive group (with the label as 1), while the normal samples were designated as the negative group (with the label as 0). True positive samples were the inner-injury fragrant pears that were recognized as inner-injury fragrant pears, true negative samples were the normal fragrant pears that were recognized as normal fragrant pears, false positive samples were the normal fragrant pears that were recognized as inner-injury fragrant pears, and false negative samples were the inner-injury fragrant pears that were recognized as normal pears.

## 3. Results

### 3.1. Electrical Parameters

The electrical parameters of the samples at three frequencies are shown in Table 1, Table 2 and Table 3. Taking Table 1 as an example, at the measuring frequency of 10 kHz, there was no discernible difference between inner injury and normal fragrant pears in terms of parallel equivalent capacitance, parallel equivalent inductance, or complex impedance. There was no discernible difference in the group of inner-injury degrees for the parallel equivalent capacitance, parallel equivalent inductance, and complex impedance. For inner injury and normal fragrant pears, there were considerable changes in the quality factor, parallel equivalent resistance, and phase angle. The quality factor, parallel equivalent resistance, and phase angle among the various levels of inner injury in fragrant pears varied significantly.

The parallel equivalent capacitance and parallel equivalent inductance of inner-injury fragrant pears were comparable to those of normal pears at all measurement frequencies (Table 1, Table 2 and Table 3). The findings demonstrated that the deformation causing the inner injury to fragrant pear would not result in an obvious change in capacitor capacitance in a parallel circuit. In this study, the detection circuit was capacitive; the inductance measured with a capacitive circuit was negative and had no practical significance. It was improper to use these two parameters to identify the inner injury of fragrant pear. The differences in quality factor, parallel equivalent resistance, and phase angle between the inner injury and normal pear were obvious. The inner-injury fragrant pear had larger quality factors, parallel equivalent resistance, and phase angles than the normal fragrant pear did. The values of the quality factors, parallel equivalent resistance, and phase angles increased with the degree of inner injury. This may be the result of alterations in fragrant pear pulp tissue brought on by recessive injury [2].

The complex impedance of the inner-injury pear was different from that of the non-destructive pear. The complex impedance value of the inner-injury pear measured at 10 kHz was greater than that of the non-destructive pear (Table 1), while that of the inner-injury pear measured at 100 kHz and 1 MHz was less than that of the non-destructive pear (Table 2 and Table 3). The measurement frequency had a significant impact on the complex impedance [38]. The influence of measurement frequency should be considered when complex impedance is used as a characteristic parameter.

### 3.2. Correlation Analysis

**Figure 2:**
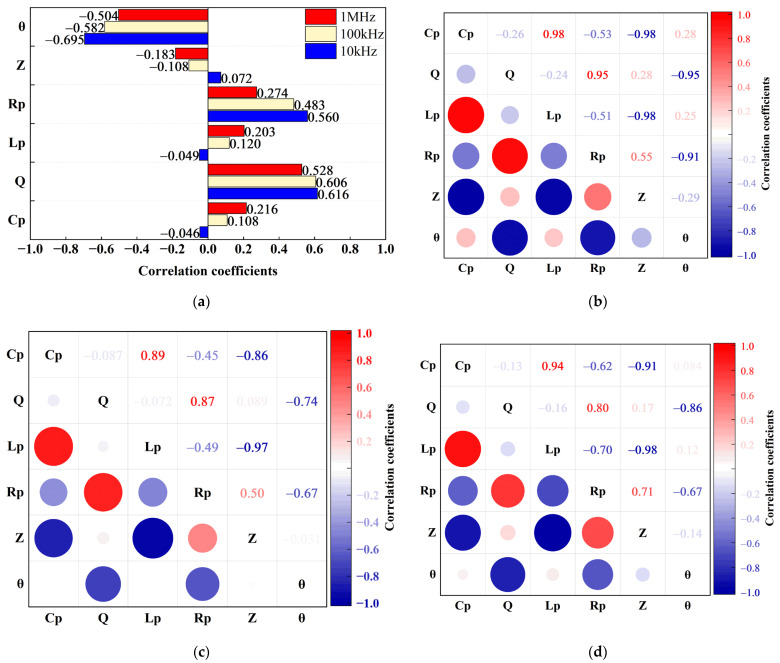
Results of the correlation analysis. (**a**) Correlation coefficients between the electrical parameters and the qualitative values; Cross-correlation coefficients among the six electrical parameters acquired at 10 kHz (**b**), 100 kHz (**c**), 1 MHz (**d**). The colors red and blue signify positive and negative correlations, respectively, and the size of the circle represents the strength of the association. The larger the circle, the stronger the correlation.

The correlation coefficient between electrical parameters and category labels was determined by the Spearman correlation analysis method was displayed in Figure 2a. Strongly correlated factors may be used as classification feature parameters. The correlation coefficients between the parallel equivalent capacitance, complex impedance, and parallel equivalent inductance, measured at three frequencies, were all less than 0.3 in absolute terms (Figure 2a), indicating that there was little relationship between these three parameters and the classification labels [33]. The absolute values of the correlation coefficients between the quality factor, parallel equivalent resistance, phase angle, and classification labels were mostly greater than 0.5 (Figure 2a), indicating that these three parameters had a certain correlation with classification labels of non-destructive and inner-injury fragrant pears, and could be used as characteristic electrical parameters to distinguish normal fragrant pears from inner-injury pears.

Figure 2b–d show the correlation coefficients between electrical parameters obtained using the Pearson correlation analysis method. The electrical parameters measured at 10 kHz and 100 kHz had strong correlations (Figure 2b,c). The correlations between quality factor, parallel equivalent resistance, and phase angle were strong, as were the correlations between parallel equivalent capacitance, complex impedance, and parallel equivalent inductance, while the correlations between the two groups of parameters were weaker. At the measurement frequency of 1 MHz, there was a high correlation among the quality factor, parallel equivalent resistance, and phase angle (Figure 2d). There was also a significant correlation between parallel equivalent resistance and parallel equivalent capacitance, complex impedance, and parallel equivalent inductance. Principal component analysis was used to reduce the dimension because there was information redundancy between electrical parameters.

### 3.3. Principal Component Analysis

Principal component analysis was performed on parallel equivalent capacitance, quality factor, parallel equivalent resistance, complex impedance, parallel equivalent inductance, and phase angle.

**Figure 3:**
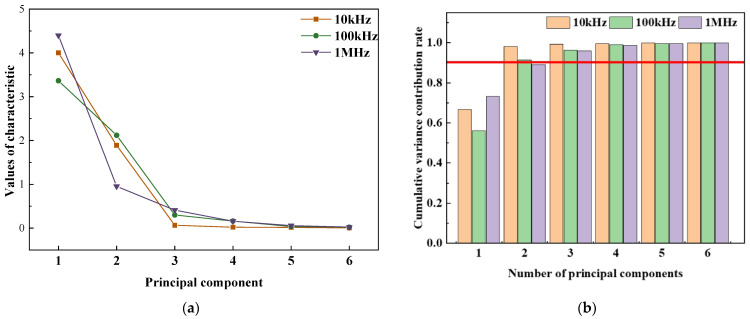
Principal component analysis: (**a**) Scree plot for the characteristic values of the principal components; (**b**) Percentage of variance for the principal components.

At the measurement frequencies of 10 kHz and 100 kHz, the characteristic values of the first two principal components were both greater than 1 (Figure 3a), and the variance cumulative contribution rate of the first two principal components exceeded 91% (Figure 3b).

The more information the principal component retained about the original variable, the larger the load value [39]. The absolute values of the characteristic load of the first principal component (PC1) and the second principal component (PC2) were similar in Table 4, indicating that PC1 and PC2 represented the information of various electrical parameters in a balanced manner [32], and the first two principal components could be used as characteristic parameters to establish classification models.

### 3.4. Modeling and Model Validation

Classification models were established using NB, KNN, and SVM to classify the inner injury and normal fragrant pears using the quality factor, parallel equivalent resistance, phase angle, and the first two principal components as characteristic parameters at three measurement frequencies. To improve the prediction performance of the models, especially the performance of the trained models on new data, 10-fold cross-validation was used to reduce the possibility of overfitting. The aim of 10-fold cross-validation was to divide the training dataset into 10 parts, taking turns to train 9 parts of them and validate 1 part of them. The average of the 10 results was used as an estimate of the algorithm’s accuracy. All the training data were involved in modeling, which could well estimate the training accuracy of the models [40].

The performance of the NB, SVM, and KNN classification models for recognition was assessed using accuracy, precision, recall, and F_1_ score. In actual production, the loss caused by inner-injury pears during storage is immeasurable. All of the inner-injury pears are expected to be identified correctly, so the effect of recall is more important.

**Figure 4:**
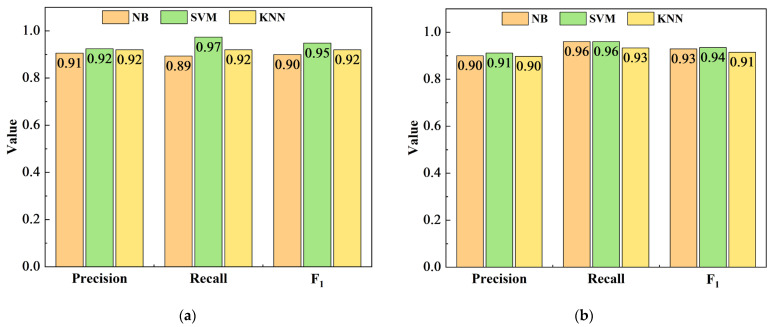
Performance comparison of Naïve Bayes (NB), Support Vector Machine (SVM), and K-Nearest Neighbor (KNN) models for the invisible injury Korla pear at the frequency of 100 kHz. (**a**) Quality factor, parallel equivalent resistance, and phase angle as the input parameters; (**b**) The first two principal components as the input parameters.

The quality factor, parallel equivalent resistance, and phase angle were used as the characteristic electrical parameters at the three measurement frequencies for the classification models. The recognition accuracy of the SVM and KNN discrimination models was superior to that of the NB model. This may be because NB is founded on the assumption that the feature parameters are independent of one another and that the redundant attributes of the feature electrical parameters will lower the prediction performance [41]. The classification models established at 10 kHz and 100 kHz were better than those of the models established at 1 MHz to identify the inner-injury pears. The SVM model, when evaluated with the confusion matrix, precision, and recall, had the best classification effect at 100 kHz, correctly identifying 19 of 25 normal pears and 73 of 75 inner-injury pears (Table 5); the recognition accuracy was 92.00%, the precision was 92.41%, the recall was 97.33%, and the F_1_ score was 0.95 (Figure 4a).

The first two principal components were used as characteristic parameters to establish classification models. The recognition accuracies of SVM classification models were superior to those of NB and KNN models at the three measurement frequencies, and the effectiveness of discriminant models established at 10 kHz and 100 kHz was superior to that established at 1 MHz in identifying the inner-injury pears. When the measurement frequencies were 10 kHz and 100 kHz, the recognition accuracy was 90.00% (Table 6). The SVM discriminant model correctly identified 70 of 75 inner-injury pears at the measurement frequency of 10 kHz, and the precision and recall were both 93.33% (Figure 4b). Under 100 kHz, the SVM discriminant model correctly identified 72 of 75 inner-injury pears (Table 6), with a precision of 91.14%, a recall of 96.00%, and an F_1_ score of 0.94 (Figure 4b). Taking into account the confusion matrix, precision, recall, and F_1_ score, the SVM model established with the measurement frequency of 100 kHz had the best discriminant effect when the first two principal components were used as the characteristic parameters.

When the measurement frequency was set to 100 kHz, the quality factor, parallel equivalent resistance, and phase angle were used as input parameters, and the constructed SVM classification model produced the best discrimination result for identifying the inner injury of fragrant pears. The quality factor, parallel equivalent resistance, and phase angle are three recommended electrical metrics for identifying inner-injury pears.

## 4. Conclusions

In this study, electrical parameters were used to detect the inner injury of the Korla fragrant pear. The parallel equivalent capacitance, quality factor, parallel equivalent inductance, parallel equivalent resistance, impedance, and phase angle of the inner-injury fragrant pears and normal pear samples were measured at three measurement frequencies. The electrical parameters of the inner-injury fragrant pear and normal fragrant pear were different. They have the potential to detect inner-injury fragrant pear. The quality factor, phase angle, and parallel equivalent resistance had significant correlations with the inner injury of fragrant pears and were used as characteristic parameters to identify the inner injury. The classification model was created using the quality factor, phase angle, and parallel equivalent resistance as typical electrical parameters, and better categorization results were achieved. With the preferred frequency of 100 kHz, the SVM model had a recognition accuracy of 92.00%, a precision of 92.41%, a recall of 97.33%, and an F_1_ score was 0.95. It was clear that it was feasible to identify the inner injury of fragrant pear with electrical parameters. This study could provide a reference for the development of an online injury recognition system based on electrical parameters.

## Figures and Tables

**Table 1:** Electrical parameters of samples measured at a measurement frequency of 10 kHz ^1^.

D (mm)	Cp (pF)	Q	Lp (H)	Rp (MΩ)	Z (MΩ)	θ (°)
0	9.005 ± 0.801 ^a^	12.777 ± 1.473 ^d^	−28.336 ± 2.514 ^a^	22.864 ± 3.988 ^d^	1.729 ± 0.156 ^a^	−85.321 ± 0.554 ^a^
3	8.949 ± 0.895 ^a^	15.605 ± 2.054 ^c^	−28.607 ± 3.07 ^a^	28.317 ± 6.247 ^c^	1.745 ± 0.187 ^a^	−86.139 ± 0.491 ^b^
5	8.917 ± 0.945 ^a^	19.69 ± 3.156 ^b^	−28.666 ± 3.209 ^a^	35.989 ± 8.826 ^b^	1.763 ± 0.19 ^a^	−86.92 ± 0.541 ^c^
7	8.882 ± 0.671 ^a^	25.108 ± 2.208 ^a^	−28.673 ± 2.228 ^a^	45.249 ± 5.511 ^a^	1.764 ± 0.139 ^a^	−87.599 ± 0.251 ^d^

^1^ The mean values ± standard deviations followed by different letters in the column differ significantly (*p* < 0.05). Cp: parallel equivalent capacitance; Q: quality factor; Lp: parallel equivalent inductance; Rp: parallel equivalent resistance; Z: impedance; θ: phase angle; D: the amount of deformation set to create inner injury.

**Table 2:** Electrical parameters of samples measured at a measurement frequency of 100 kHz ^1^.

D (mm)	Cp (pF)	Q	Lp (mH)	Rp (MΩ)	Z (kΩ)	θ (°)
0	8.171 ± 0.848 ^a^	18.324 ± 2.64 ^d^	−314.777 ± 52.355 ^b^	3.679 ± 1.295 ^d^	192.093 ± 32.768 ^a^	−86.524 ± 1.216 ^d^
3	8.313 ± 0.785 ^a^	22.258 ± 3.153 ^c^	−307.617 ± 31.131 ^b,a^	4.348 ± 0.985 ^c^	186.894 ± 20.451 ^a^	−87.248 ± 0.402 ^c^
5	8.385 ± 0.904 ^a^	28.247 ± 4.919 ^b^	−305.701 ± 35.003 ^b,a^	5.35 ± 1.293 ^b^	187.495 ± 20.534 ^a^	−87.821 ± 0.318 ^b^
7	8.44 ± 0.803 ^a^	34.766 ± 3.634 ^a^	−300.051 ± 23.581 ^a^	6.547 ± 0.86 ^a^	184.619 ± 14.479 ^a^	−88.251 ± 0.237 ^a^

^1^ The mean values ± standard deviations followed by different letters in the column differ significantly (*p* < 0.05). Cp: parallel equivalent capacitance; Q: quality factor; Lp: parallel equivalent inductance; Rp: parallel equivalent resistance; Z: impedance; θ: phase angle; D: the amount of deformation set to create inner injury.

**Table 3:** Electrical parameters of samples measured at a measurement frequency of 1 MHz ^1^.

D (mm)	Cp (pF)	Q	Lp (mH)	Rp (kΩ)	Z (kΩ)	θ (°)
0	8.114 ± 0.792 ^c^	13.344 ± 1.789 ^d^	−3.167 ± 0.489 ^c^	268.599 ± 84.7 ^b^	19.195 ± 3.065 ^a^	−85.536 ± 0.579 ^a^
3	8.321 ± 0.767 ^b,c^	14.664 ± 1.753 ^c^	−3.061 ± 0.316 ^b,c^	285.794 ± 57.86 ^b^	18.598 ± 1.759 ^b,a^	−85.885 ± 0.566 ^b^
5	8.52 ± 0.893 ^a,b^	16.536 ± 1.848 ^b^	−3.006 ± 0.335 ^a,b^	314.699 ± 64.43 ^a^	18.361 ± 1.955 ^b^	−86.342 ± 0.455 ^c^
7	8.706 ± 0.646 ^a^	18.428 ± 1.778 ^a^	−2.92 ± 0.226 ^a^	337.754 ± 47.079 ^a^	17.864 ± 1.366 ^b^	−86.79 ± 0.393 ^d^

^1^ The mean values ± standard deviations followed by different letters in the column differ significantly (*p* < 0.05). Cp: parallel equivalent capacitance; Q: quality factor; Lp: parallel equivalent inductance; Rp: parallel equivalent resistance; Z: impedance; θ: phase angle; D: the amount of deformation set to create inner injury.

**Table 4:** The loading value of electrical parameters in the principal component at three measurement frequencies.

Parameter	10 kHz		100 kHz		1 MHz	
	PC1	PC2	PC1	PC2	PC1	PC2
Cp	−0.41914	0.38758	−0.45083	0.31033	−0.4265	0.36479
Q	0.36809	0.48569	0.29302	0.54503	0.35387	0.56292
Lp	−0.41294	0.40082	−0.47113	0.32675	−0.41169	0.40611
Rp	0.45025	0.30226	0.45876	0.33487	0.45276	0.22491
Z	0.42464	−0.37592	0.4694	−0.31681	0.446	−0.32307
θ	−0.36779	−0.46963	−0.24108	−0.53606	−0.34566	−0.47975

PC1: the first principal component; PC2: the second principal component. Cp: parallel equivalent capacitance; Q: quality factor; Lp: parallel equivalent inductance; Rp: parallel equivalent resistance; Z: impedance; θ: phase angle.

**Table 5:** The classification results for the invisible pear using the trained models with the quality factor, parallel equivalent resistance, and phase angle as the input parameter.

	Actual	10 kHz			100 kHz			1 MHz		
		Prediction		ACC	Prediction		ACC	Prediction		ACC
Model		0	1		0	1		0	1	
NB	0 (*N* = 25)	24	1	86.00%	18	7	85.00%	15	10	75.00%
	1 (*N* = 75)	13	62		8	67		12	63	
SVM	0 (*N* = 25)	18	7	88.00%	19	6	92.00%	16	9	83.00%
	1 (*N* = 75)	5	70		2	73		8	67	
KNN	0 (*N* = 25)	20	5	89.00%	19	6	88.00%	16	9	86.00%
	1 (*N* = 75)	6	69		6	69		5	70	

0: normal pear; 1: invisible injury pear.

**Table 6:** The classification results for the invisible pear using the trained models with the first two principal components as the input parameter.

		10 kHz			100 kHz			1 MHz		
		Prediction		ACC	Prediction		ACC	Prediction		ACC
Model	Actual	0	1		0	1		0	1	
NB	0 (*N* = 25)	14	11	85.00%	17	8	89.00%	12	13	81.00%
	1 (*N* = 75)	4	71		3	72		6	69	
SVM	0 (*N* = 25)	20	5	90.00%	18	7	90.00%	13	12	82.00%
	1 (*N* = 75)	5	70		3	72		6	69	
KNN	0 (*N* = 25)	19	6	90.00%	17	8	87.00%	16	9	81.00%
	1 (*N* = 75)	4	71		5	70		10	65	

0: normal pear; 1: invisible injury pear.

## Data Availability

The data presented in this study are available on request from the corresponding author. The data are not publicly available, due to the request for funding scientific research projects.
